# Cross-Sectional Relationship between Carotid-Femoral Pulse Wave Velocity and Biomarkers in Vascular-Related Diseases

**DOI:** 10.1155/2020/6578731

**Published:** 2020-05-23

**Authors:** Jinbo Liu, Kuanting Wang, Huan Liu, Hongwei Zhao, Wei Huang, Na Zhao, Lihong Li, Hongyu Wang

**Affiliations:** ^1^Department of Vascular Medicine, Peking University Shougang Hospital, Beijing 100144, China; ^2^Department of Rheumatology and Immunology, Peking University Shougang Hospital, Beijing 100144, China

## Abstract

**Objectives:**

The present study was done to investigate the relationship between carotid-femoral pulse wave velocity (CFPWV) and biomarkers such as homocysteine (Hcy), N-terminal pro-brain natriuretic peptide (NT-proBNP), and urine albumin (microalbumin) (UAE) in vascular-related diseases.

**Methods:**

656 subjects were enrolled into our study. There were 377 patients with hypertension, 231 with coronary heart disease, 154 with diabetes mellitus, and 186 healthy subjects. They were divided into four groups according to the number of suffered diseases: group 1 had only one of three diseases, group 2 had two, and group 3 had all of three diseases. CFPWV was measured by Complior apparatus.

**Results:**

CFPWV was significantly higher in group 3 than in the healthy group, group 1, and group 2 (12.71 ± 2.38 vs 10.11 ± 2.28, 10.70 ± 2.12, and 11.92 ± 2.55, all *p* < 0.05). The level of Hcy was significantly higher in group1, group 2, and group 3 than in healthy subjects, respectively. Levels of Log NT-proBNP and Log UAE were significantly higher in group 3 than in group 1 (2.27 ± 0.4 vs 2.10 ± 0.4, 1.00 ± 0.65 vs 0.68 ± 0.56, both *p* < 0.05). Positive correlation between CFWV and Hcy, Log UAE, and Log NT-proBNP was found in the entire study group (*r* = 0.109, 0.196, and 0.119, all *p* < 0.05). Multivariate analysis showed that pulse pressure, age, fasting plasma glucose, diastolic blood pressure, body mass index, and Log UAE were independent associating factors of CFPWV in all subjects (*β* = 0.334, *p* < 0.001; *β* = 0.333, *p* < 0.001; *β* = 0.126, *p*=0.004; *β* = 0.137, *p*=0.003; *β* = −0.142, *p*=0.002; and *β* = 0.098, *p*=0.031).

**Conclusions:**

CFPWV was significantly higher in subjects with hypertension, CHD, and DM. There was correlation between CFPWV and biomarkers such as NT-proBNP, Hcy, and urine albumin (microalbumin).

## 1. Introduction

Coronary heart disease (CHD), hypertension, and diabetes mellitus (DM) are the most serious vascular-related diseases threatening the human's life. Arteriosclerosis is the basic pathophysiological change of these diseases, and it can be measured by arterial stiffness. A recent study showed that arterial stiffness is a strong predictor of future cardiovascular events and all-cause mortality [1]. Carotid-femoral pulse wave velocity (CFPWV) is considered as a golden evaluation index of arterial stiffness suggested by ESH/ESC [2]. A recent study showed that the level of CFPWV was higher in DM subjects with higher HbA1c [3]. A recent review showed that PWV was a marker of cognitive impairment in the elderly subjects [4]. Our previous studies also showed that CFPWV was positively correlated with pulse pressure and it was increased in hypertension patients with left ventricular hypertrophy [5, 6]. And CFPWV was significantly higher in healthy subjects with positive family history of hypertension [7].

As we know, hyperglycemia and hyperlipidemia are traditional risk factors leading to the development of vascular-related diseases. However, the incidence of vascular-related diseases was still increasing after glucose and lipid control. So, there might be other risk factors involving the mechanism of vascular-related diseases or inappropriate therapeutic targets during treatment. Recent studies showed that plasma biomarkers such as homocysteine (Hcy), N-terminal pro-brain natriuretic peptide (NT-proBNP), and urine albumin (microalbumin) (UAE) have involved the pathophysiological development of arteriosclerosis and could be predictors of future cardiovascular events. For example, chronic hyperhomocysteinemia contributed to coronary artery disease by inhibiting dysfunction of the coronary artery endothelium [8]. And our previous study showed that arterial stiffness was positively correlated with NT-proBNP in hypertension subjects with CHD [9]. UAE could be a marker of microvascular lesion, and it increased significantly with the severity of hypertension [10]. The levels of biomarkers could reflect the severity of vascular-related diseases. In addition, biomarker research could provide some information for the mechanism of vascular-related diseases and therapy target.

As shown above, CHD, hypertension, and DM are the most serious vascular-related diseases threatening the human's life. However, many patients were not only suffering one kind of vascular-related diseases but also suffering some of them, such as one patient might suffer hypertension, DM, and CHD simultaneously in our routine clinical examination. And this is the real world about patients. The levels or relationship between CFPWV and biomarkers could be different in patients with one or more vascular-related diseases. So, our present was carried out to investigate the relationship between CFPWV and biomarkers in these subjects, to provide some information for the mechanism and prevention of arteriosclerosis.

## 2. Materials and Methods

### 2.1. Subjects

This was a retrospective study. 656 subjects (M/F, 272/384) from the Department of Vascular Medicine from January 2012 to December 2013 were enrolled into our study. Subjects with stroke, arteriosclerosis obliterans, heart failure, renal function impairment, liver function impairment, systemic inflammatory diseases, infectious disease, or cancer were excluded. 186 subjects were outpatient for health examination population without the diseases of hypertension, CHD, and DM. The rest of 470 subjects were inpatient. And of these 470 subjects, there were 377 patients with hypertension, 231 with CHD, and 154 with DM.

We divided our study group into four groups according to the numbers of suffered diseases: healthy subjects (*N* = 186, without the diseases of hypertension, CHD, and DM), group 1 (*N* = 237, with one of the diseases of hypertension, CHD, and DM), group 2 (*N* = 174, with two of the diseases of hypertension, CHD, and DM), and group 3 (*N* = 59, with all diseases of hypertension, CHD, and DM).

Hypertension was defined as blood pressure measurement ≥140/90 mmHg in three occasions at rest or subjects with known cases of diagnosed hypertension before and taking antihypertensive drugs at present. CHD was defined as the narrowing (>75%) or blockage of coronary artery diagnosed by angiography. DM was defined according to the 75 g oral glucose tolerance test (OGTT). All participants gave their written informed consent. This study was approved by the Ethics Committee of Peking University Shougang Hospital, China.

### 2.2. The Assessment of ABI

ABI was recorded using a VaseraVS-1000 vascular screening system (Fukuda Denshi, Tokyo, Japan) with the participant resting in a supine position. ECG electrodes were placed on both wrists, a microphone for detecting heart sounds was placed on the sternum, and cuffs were wrapped around both the arms and ankles. After automatic measurements, obtained data were analyzed by software, and the value of ABI was obtained automatically.

### 2.3. Pulse Wave Velocity Measurement

Arterial stiffness was evaluated by measuring automatic PWV using the Complior apparatus. The basic principle of PWV assessment is that pressure pulse generated by ventricular ejection is propagated along the arterial system at a speed determined by elasticity of the arterial wall. Knowing the distance and pulse transit time, the velocity can be calculated. Patients were placed in recumbent position and, after a 10-minute rest, underwent PWV measurement, and CFPWV was obtained automatically [11].

### 2.4. Laboratory Measurements

Blood samples were drawn from an antecubital vein in the morning after overnight fasting and collected into vacuum tubes containing EDTA for the measurement of plasma lipid and lipoprotein levels. Fasting plasma glucose (FPG), total cholesterol (TC), high-density lipoprotein cholesterol (HDL-C), triglyceride (TG), and homocysteine levels were analyzed by colorimetric enzymatic assays with the use of an autoanalyzer (HITACHI-7170, Hitachi, Tokyo, Japan) at the central chemistry laboratory of the Peking University Shougang Hospital. Low-density lipoprotein cholesterol (LDL-C) levels were calculated. Urine albumin (microalbumin) excretion (UAE) was determined by the 12-hour overnight urine samples calculated from the urine albumin concentration, urine volume, and collection time at the central chemistry laboratory of the Peking University Shougang Hospital. The level of NT-proBNP was measured using the method of Biodirectional lateral flow immunoassay (ReLIA) according to the procedure.

### 2.5. Statistical Analysis

SPSS was used in the statistical analysis. The one-sample Kolmogorov–Smirnov test was used to test the normal distribution. The levels of NT-proBNP and UAE were not in normal distribution, so we made log transformation of NT-proBNP and UAE. The differences between groups were analyzed by one-way ANOVA and least-significant difference (LSD). Proportions were analyzed by *χ*^2^-test. Correlation coefficient was done to find linear relation between different variables using Pearson correlation coefficient. Partial correlation analysis was also used in the present study. Multivariate linear regressions (stepwise) were used to estimate the coefficients of the linear equation, involving independent variables including age, BMI, SBP, DBP, pulse pressure, FPG, UA, creatinine, TC, HDL-C, LDL-C, TG, Log NT-proBNP, Hcy, and Log UAE that affected the value of CFPWV. And the F value of multivariate linear regressions and Fa (*k*, *n*−*k*−1) value were also calculated to judge whether it was valid or not. Values were shown as mean ± SD unless stand otherwise. In addition, the one-sample Kolmogorov–Smirnov test showed that Log UAE was not in normal distribution in group 2 (*p*=0.020), with normal distribution in the healthy group, group 1, and group 3 (all *p* > 0.05). In addition, Log BNP was in normal distribution in all subgroups. And Log UAE and Log NT-proBNP were not in normal distribution in the CHD group and non-CHD group. So, Me (*Q*1–*Q*3) for Log UAE and Log NT-proBNP was presented. *p* < 0.05 (2-tailed) was considered statistically significant.

## 3. Results

### 3.1. Clinical Characteristics of the Study Participants

The baseline clinical characteristics of study participants are shown in Table 1. As shown in Table 1, 237 patients had only one of these three vascular-related diseases, 174 patients had two of these three vascular-related diseases, 59 patients had all of these three diseases, and 186 subjects with none of these three vascular-related diseases.

Our results showed that with the increasing numbers of suffered vascular-related diseases, the level of CFPWV was increasing. Similar results were also found in the parameters of biomarkers of Hcy, Log NT-ProBNP, and Log UAE. In addition, the levels of ABI and HDL-C were decreasing with the increasing numbers of suffered diseases.

In addition, CHD was accompanied with structural atherosclerotic change resulted from hypertension and DM. And we divided these subjects into two groups according to with or without CHD. As shown in Table 2, the levels of CFWV, uric acid, Hcy, Log NT-ProBNP, and Log UAE were significantly higher in the CHD group. The level of LDL-C was lower in the CHD group, according to higher usage of lipid-lowering agents.

### 3.2. Pearson Correlations between CFPWV and Metabolic Markers

Next, we investigated the Pearson correlations between biomarkers and the vascular function index in the entire group and in subgroups. Our results showed that Log NT-proBNP was positively correlated with CFPWV in the entire study group (*r* = 0.119, *p* < 0.05) and nonhealthy group (*r* = 0.164, *p* < 0.05). There was negative correlation between Log NT-proBNP and RABI and LABI in the entire study group (*r* = −0.171 and −0.145, both *p* < 0.05, respectively) and nonhealthy group (*r* = −0.197 and −0.206, both *p* < 0.05, respectively). There was positive correlation between Hcy and CFPWV in the entire study group (*r* = 0.109, *p* < 0.05) and nonhealthy group (*r* = 0.093, *p*=0.050). In addition, our present study showed that there was positive correlation between Log UAE and CFPWV in the entire study group (*r* = 0.196, *p* < 0.05) and nonhealthy group (*r* = 0.209, *p* < 0.050).

As shown above, there was significant difference about age, drug usage, BMI, blood pressure, FPG, uric acid, and lipids between these groups. So, we made partial correlation analysis next. And our present result showed that there was positive correlation between Log UAE and CFPWV in the entire study group (*r* = 0.105, *p*=0.050) after adjusting for age, BMI, FPG, blood pressure, uric acid, and lipids without significant difference between Log NT-proBNP, Hcy, and CFPWV after adjusting.

As shown in Table 3, subgroup Pearson correlation analysis showed that there was positive correlation between CFPWV and age, SBP, and pulse pressure in all subgroups. And significant negative correlation between CFPWV and RABI (*r* = −0.315, *p*=0.015) was found in group 3, with negative correlation tendency between CFPWV and LABI (*r* = −0.239, *p*=0.069).

### 3.3. Multivariate Regression Analysis

Multivariate linear regressions (stepwise) were used to estimate the coefficients of the linear equation, involving independent variables including age, BMI, SBP, DBP, pulse pressure, FPG, UA, creatinine, TC, HDL-C, LDL-C, TG, Log NT-proBNP, Hcy, and Log UAE that affected the value of CFPWV. The *F* value of multivariate linear regressions was 36.881 (*p* < 0.001), and the Fa (0.05, 15,656) value was 1.68, so it was valid. Our results showed that pulse pressure, age, FPG, DBP, BMI, and Log UAE were independent associating factors of CFPWV in all subjects (*β* = 0.334, *p* < 0.001; *β* = 0.333, *p* < 0.001; *β* = 0.126, *p*=0.004; *β* = 0.137, *p*=0.003; *β* = −0.142, *p*=0.002; and *β* = 0.098, *p*=0.031, respectively, Tables 4 and 5).

## 4. Discussion

Our present study showed that CFPWV was significantly higher in subjects with hypertension, CHD, and DM, with decreasing level of ABI. And there was correlation between CFPWV and biomarkers such as Log NT-proBNP, Hcy, and Log UAE.

CHD, hypertension, and DM are the most serious vascular-related diseases with similar pathophysiological changes such as arteriosclerosis. Arteriosclerosis could be evaluated by arterial stiffness, which is measured by CFPWV suggested by ESH/ESC [2]. A previous study showed that aortic PWV was an independent predictor of coronary heart disease and aortic PWV also provided additional predictive value above cardiovascular risk factors [12]. A prospective study showed that carotid and femoral stiffness indices were independently associated with incident cardiovascular events and all-cause mortality [13]. Mulders et al. [14] found that patients with premature CHD and their first-degree relatives had higher PWV compared with controls, independent of other risk factors, and Kingwell et al. reported that large artery stiffness was a major determinant for myocardial ischemic threshold in patients with coronary artery disease [15]. A recent study showed that aortic stiffness was an independent predictor of incident mild cognitive impairment and a potentially modifiable risk factor for clinical cognitive impairment and dementia [16]. These studies confirmed the role of PWV in the prediction of vascular-related events.

However, many patients were not suffering only one kind of vascular-related disease but also suffering some of them, such as one patient might be found to have hypertension, DM, and CHD simultaneously in our routine clinical examination. And this is the real world about patients. And our present study showed that CFPWV was increasing with the increasing number of suffered diseases, indicating that multiple diseases could worsen arterial stiffness. So, arterial stiffness should be more emphasized in order to reduce vascular-related events in future clinical therapy.

Biomarkers of vascular-related diseases such as homocysteine (Hcy), N-terminal pro-brain natriuretic peptide (NT-proBNP), and urine albumin (microalbumin) (UAE) have involved the pathophysiological development of arteriosclerosis. Hyperhomocysteinemia (HHcy) has been considered as an independent risk factor for atherosclerosis [17]. And a recent study showed that plasma total homocysteine was an independent risk factor for stroke [18]. Our present study showed that Hcy was increasing the increasing number of suffered diseases, and positive correlation was found between Hcy and CFPWV. NT-proBNP is a controregulatory hormone associated with cardiac remodeling such as left ventricular hypertrophy and systolic/diastolic dysfunction. In addition to the role of NT-proBNP in the disease of heart failure, a recent study showed that NT-proBNP is a more significant prognostic factor for cardiovascular mortality [19]. In addition, our previous study also showed the similar result about the relationship between arterial stiffness and NT-proBNP in hypertension subjects with coronary artery disease [9].

Microalbuminuria was an early indicator of impaired renal function caused by hypertension, and the UAE increased significantly with the severity of hypertension [20]. A recent study showed that low levels of albuminuria were associated with increased risk of incident hypertension and CHD mortality at follow-up [21]. And microalbuminuria was significantly correlated to diabetes duration [22]. A recent review showed that microalbuminuria might not simply be regarded as a risk predictor but become itself an independent target for treatment of cardiovascular events [23, 24]. Metabolic syndrome and all its components were associated with the presence of microalbuminuria in a health checkup population in China [25]. Our present study showed that level of Log UAE was increasing with the number of suffering diseases increasing, and there was positive correlation between Log UAE and CFPWV. In addition, multivariate linear regression analysis showed that Log UAE was an independent associating factor of CFPWV. In addition, DBP and FPG were independent associating factors of CFPWV, and Log UAE was an indicator of vascular-related diseases complications of renal dysfunction, this might indicate the relationship between early renal dysfunction and CFPWV. Andfurther study should be done in future. In addition, similar to the result of the present study, Kong et al. found that arterial stiffness increases the risk of proteinuria in a Chinese population-based cohort [26].

Our present study showed that arterial stiffness was increasing with the number of suffered vascular-related diseases increasing, with the levels of some biomarkers increasing. As shown above, arterial stiffness and biomarkers could both be the predictors of future cardiovascular events. So, the predictive value might be improved by combination of arterial stiffness and biomarkers together. A recent study showed that aortic PWV improves risk prediction when added to standard risk factors [27]. Blankenberg et al. showed that combing traditional risk factors with NT-proBNP can provide the best clinical prediction of recurrent cardiovascular events in the secondary-prevention population [28]. In addition, the MORGAM study confirmed that adding N-terminal pro-brain natriuretic peptide, C-reactive protein, and sensitive troponin I to a conventional risk model can improve 10-year risk estimation for cardiovascular events [29]. So, we might collect some arterial function indexes and some biomarkers together, called early vascular lesion detection technology, to provide some information for the complete and accurate evaluation of one patient. We established the early vascular lesion detection system and published the first guideline in 2005 [30], the Chinese Guideline for Early Vascular Disease Detection (first report). This guideline was renewed in 2011 as the Chinese Guideline for Early Vascular Disease Detection (2011, second report) [31].

A major limitation of our study was its cross-sectional design; another limitation was that the subjects' numbers of each group were not balanced. In addition, some patients with CHD or hyperlipids had statins when enrolled into our present study, so the level of LDL-C was decreasing in subjects with two or more kinds of diseases. In addition, our previous research [32] about relationship between the cardio-ankle vascular index (CAVI) and biomarkers in vascular-related diseases showed that age, BMI, Log UAE, and DBP were independent associating factors of the cardio-ankle vascular index in all subjects. Both PWV and CAVI are the evaluation index of arterial stiffness with different points of focus [33]. However, our present study might provide some information about relationship between arterial stiffness and biomarkers. So, more studies and analysis should be investigated in future (Table 5).

In conclusion, our present study showed that CFPWV was significantly higher in subjects with hypertension, CHD, and DM with decreasing level of ABI. And there was correlation between CFPWV and biomarkers such as NT-proBNP, Hcy, and UAE. And a vascular lesion evaluation system including arterial function and biomarkers could be founded in future.

## Figures and Tables

**Table 1:** Clinical characteristics in different groups.

Characteristics	Healthy (*N* = 186)	Group 1 (*N* = 237)	Group 2 (*N* = 174)	Group 3 (*N* = 59)	*pvalue*
Age (year）	56.0 ± 10.4	60.0 ± 10.0^*∗*^	65.0 ± 11.5^*∗#*^	67.2 ± 11.7^*∗#*^	<0.001
Male/female	74/112	97/140	78/96	23/36	0.752
CCB (%)	0	18.1^*∗*^	29.3^*∗#*^	49.2^*∗#*^%	<0.001
ACEI/ARB (%)	0	17.3^*∗*^	28.7^*∗#*^	44.0^*∗#*^%	<0.001
B-blocker (%)	0	13.5^*∗*^	31.0^*∗#*^	33.9^*∗#*^	<0.001
Antidiabetic agent (%)	0	4.6^*∗*^	21.2^*∗#*^	72.9^*∗#*^%	<0.001
Lipid-lowering					
agent (%)	0	27.4^*∗*^	41.4^*∗#*^	54.2^*∗#*^%	<0.001
Smoking (%)	24.7	27.8	32.7	27.1	0.344
BMI (kg/m^2^)	24.38 ± 3.46	25.65 ± 3.43^*∗*^	26.07 ± 3.40^*∗*^	26.09 ± 3.66^*∗*^	<0.001
CFPWV (m/s)	10.11 ± 2.28	10.70 ± 2.12^*∗*^	11.92 ± 2.55^*∗#*^	12.71 ± 2.38^*∗#*^%	<0.001
RABI	1.130 ± 0.10	1.125 ± 0.08	1.098 ± 0.11^*∗#*^	1.058 ± 0.12^*∗#*^%	<0.001
LABI	1.116 ± 0.12	1.122 ± 0.09	1.095 ± 0.11^#^	1.083 ± 0.15^*∗#*^	0.019
SBP (mmHg)	128.8 ± 16.2	139.0 ± 19.4^*∗*^	145.0 ± 20.3^*∗*^	145.0 ± 20.0^*∗#*^	<0.001
DBP (mmHg)	80.7 ± 9.5	84.9 ± 12.0^*∗*^	86.3 ± 11.2^*∗*^	83.6 ± 11.3	<0.001
Pulse pressure (mmHg)	48.1 ± 10.6	54.0 ± 13.5^*∗*^	58.7 ± 16.0^*∗#*^	61.4 ± 14.6^*∗#*^	<0.001
FPG (mmol/L)	5.50 ± 0.85	5.69 ± 1.21	6.31 ± 1.92^*∗#*^	7.44 ± 2.53^*∗#*^%	<0.001
UA (*μ*mol/L)	298.2 ± 71.8	310.9 ± 80.7	321.9 ± 79.3^*∗*^	314.4 ± 91.7	0.043
Creatinine (*μ*mol/L)	63.2 ± 13.5	66.5 ± 22.0	68.4 ± 19.7	68.3 ± 19.8	0.055
TC (mmol/L)	5.18 ± 1.10	4.95 ± 1.11	4.44 ± 1.15^*∗#*^	4.58 ± 1.27^*∗#*^	**<0.001**
HDL-C (mmol/L)	1.33 ± 0.35	1.25 ± 0.31^*∗*^	1.14 ± 0.27^*∗#*^	1.11 ± 0.27^*∗#*^	<0.001
LDL-C (mmol/L)	3.25 ± 0.89	3.05 ± 0.89	2.59 ± 0.86^*∗#*^	2.72 ± 0.84^*∗#*^	<0.001
TG (mmol/L)	1.72 ± 1.29	1.84 ± 1.56	1.77 ± 1.80	1.96 ± 2.32	0.751
HCY (*μ*mol/L)	11.57 ± 5.90	13.82 ± 8.21^*∗*^	15.27 ± 7.21^*∗*^	15.87 ± 8.08^*∗*^	<0.001
Log NT-proBNP	1.84–2.27	1.89–2.33	2.04–2.55^*∗#*^	1.92–2.57^*∗#*^	<0.001
Log UAE	0.36–0.76	0.40–0.93	0.54–1.03^*∗#*^	0.57–1.49^*∗#*^	<0.001

*p* values were calculated using analysis of one-way ANOVA. Least-significant difference (LSD) was used to do comparison analysis between groups, marked with ∗, #, and %. ^*∗*^vs healthy, *p* < 0.05; ^#^vs group 1, *p* < 0.05; % vs group 2, *p* < 0.05; group 1, with one of the diseases of hypertension, CHD, and DM; group 2, with two of the diseases of hypertension, CHD, and DM; group 3: with all the diseases of hypertension, CHD, and DM; CHD, coronary heart disease; DM, diabetes mellitus; BMI, body mass index; CFPWV, carotid-femoral pulse wave velocity; RABI, right ankle brachial index; LABI, left ankle brachial index; SBP, systolic blood pressure; DBP, diastolic blood pressure; FPG, fasting plasma glucose; UA, uric acid; TC, cholesterol; LDL-C, low-density lipoprotein cholesterol; HDL-C, high-density lipoprotein cholesterol; TG, triglycerides; HCY, homocysteine; UAE, urine albumin (microalbumin) excretion; CCB, calcium channel blocker; ACEI, angiotensin-converting enzyme inhibitors; ARB, angiotensin receptor blocker.

**Table 2:** Clinical characteristics in different groups.

Characteristics	Non-CHD group (*N* = 425)	CHD group (*N* = 231)	*p*
Age (year）	58.3 ± 10.7	65.3 ± 11.3	<0.001
Male/female	168/257	104/127	0.173
Hypertension (%)	49.8	73.5	<0.001
DM (%)	18.6	32.5	<0.001
CCB (%)	13.2	29.4	<0.001
ACEI/ARB (%)	12.7	28.1	<0.001
B-blocker (%)	7.8	43.6	<0.001
Antidiabetic agent (%)	9.2	22.9	<0.001
Lipid-lowering	23.1	46.3	<0.001
Agent (%)			
Smoking (%)	32.2	36.4	0.285
BMI (kg/m^2^)	25.18 ± 3.46	25.94 ± 3.57	0.008
CFPWV (m/s)	10.72 ± 2.37	11.63 ± 2.52	<0.001
RABI	1.125 ± 0.099	1.091 ± 0.108	<0.001
LABI	1.116 ± 0.107	1.099 ± 0.119	0.076
SBP (mmHg)	136.6 ± 19.3	141.2 ± 20.0	0.004
DBP (mmHg)	84.1 ± 11.0	83.6 ± 11.8	0.574
Pulse pressure (mmHg)	52.4 ± 13.4	57.6 ± 15.2	<0.001
FPG (mmol/L)	5.82 ± 1.32	6.21 ± 2.02	0.010
UA (*μ*mol/L)	304.6 ± 76.5	321.5 ± 83.3	0.010
Creatinine (*μ*mol/L)	65.3 ± 19.3	67.9 ± 18.9	0.089
TC (mmol/L)	5.02 ± 1.17	4.52 ± 1.10	<0.001
HDL-C (mmol/L)	1.28 ± 0.33	1.14 ± 0.27	<0.001
LDL-C (mmol/L)	3.11 ± 0.92	2.65 ± 0.82	<0.001
TG (mmol/L)	1.77 ± 1.40	1.86 ± 2.05	0.496
HCY (*μ*mol/L)	12.73 ± 7.18	15.58 ± 7.68	<0.001
Log NT-proBNP	1.89–2.31	1.99–.57	<0.001
Log UAE	0.40–0.91	0.47–0.99	0.028

BMI, body mass index; CFPWV, carotid-femoral pulse wave velocity; RABI, right ankle brachial index; LABI, left ankle brachial index; SBP, systolic blood pressure; DBP, diastolic blood pressure; FPG, fasting plasma glucose; UA, uric acid; TC, cholesterol; LDL-C, low-density lipoprotein cholesterol; HDL-C, high-density lipoprotein cholesterol; TG, triglycerides; HCY, homocysteine; UAE, urine albumin (microalbumin) excretion; CCB, calcium channel blocker; ACEI, angiotensin-converting enzyme inhibitors; ARB, angiotensin receptor blocker.

**Table 3:** Pearson correlations between CFPWV and metabolic markers in each subgroup.

Characteristics	Healthy (*N* = 186)		Group 1 (*N* = 237)		Group 2 (*N* = 174)		Group 3 (*N* = 59)	
	*r*	*p*	*r*	*p*	*r*	*p*	*r*	*p*
Age (year)	0.451	<0.001	0.327	<0.001	0.457	<0.001	0.392	0.002
BMI (kg/m^2^)	−0.114	0.126	−0.059	0.370	−0.033	0.668	−0.228	0.083
RABI	−0.006	0.934	−0.063	0.337	−0.250	0.001	−0.315	0.015
LABI	0.069	0.349	−0.152	0.019	−0.386	<0.001	−0.239	0.069
SBP (mmHg)	0.357	<0.001	0.302	<0.001	0.476	<0.001	0.480	<0.001
DBP (mmHg)	0.122	0.098	0.166	0.011	0.224	0.003	0.003	0.983
Pulse pressure (mmHg)	0.439	<0.001	0.285	<0.001	0.448	<0.001	0.574	<0.001
FPG (mmol/L)	0.091	0.219	0.068	0.295	0.071	0.353	−0.031	0.818
UA (*μ*mol/L)	0.017	0.816	0.286	<0.001	−0.156	0.041	−0.061	0.646
Creatinine (*μ*mol/L)	0.171	0.021	0.165	0.011	0.190	0.012	0.103	0.440
TC (mmol/L)	0.077	0.296	−0.042	0.527	−0.066	0.388	0.048	0.719
HDL-C (mmol/L)	0.032	0.668	−0.077	0.240	0.052	0.496	0.239	0.071
LDL-C (mmol/L)	−0.029	0.696	−0.079	0.231	−0.038	0.621	−0.020	0.880
TG (mmol/L)	0.074	0.318	0.095	0.147	−0.187	0.014	0.001	0.994
HCY (*μ*mol/L)	−0.034	0.650	0.143	0.033	0.045	0.571	−0.172	0.205
Log NT-proBNP	0.155	0.057	−0.004	0.959	0.224	0.011	0.193	0.175
Log UAE	0.068	0.376	−0.101	0.139	0.102	0.210	−0.181	0.181

Group 1, with one of the diseases of hypertension, CHD, and DM; group 2, with two of the diseases of hypertension, CHD, and DM; group 3: with all the diseases of hypertension, CHD, and DM; CHD, coronary heart disease; DM, diabetes mellitus; BMI, body mass index; CFPWV, carotid-femoral pulse wave velocity; RABI, right ankle brachial index; LABI, left ankle brachial index; SBP, systolic blood pressure; DBP, diastolic blood pressure; FPG, fasting plasma glucose; UA, uric acid; TC, cholesterol; LDL-C, low-density lipoprotein cholesterol; HDL-C, high-density lipoprotein cholesterol; TG, triglycerides; HCY, homocysteine; UAE, urine albumin (microalbumin) excretion.

**Table 4:** Multiple linear regression analysis for the relationship between CFPWV and study variables among the entire study group, independent associating factors of CFPWV in all subjects.

	Unstandardized *β*	95% CI for *β*	Std. error	Standardized *β*	*t*	*p* value
Constant	1.786	(−0.681, 4.253)	1.254	—	1.424	
Pulse pressure	0.057	(0.041, 0.074)	0.008	0.334	6.930	<0.001
Age	0.074	(0.053, 0.094)	0.010	0.333	7.107	<0.001
FPG	0.188	(0.060, 0.315)	0.065	0.126	2.899	0.004
DBP	0.032	(0.011, 0.053)	0.011	0.137	2.996	0.003
BMI	−0.104	(−0.168, −0.039)	0.033	−0.142	−3.152	0.002
Log UAE	0.491	(0.046, 0.936)	0.226	0.098	2.170	0.031

CFPWV, carotid-femoral pulse wave velocity; BMI, body mass index; DBP, diastolic blood pressure; FPG, fasting plasma glucose; UAE, urine albumin (microalbumin) excretion; CI, confidence interval; SBP, systolic blood pressure; UA, uric acid; TC, cholesterol; LDL-C, low-density lipoprotein cholesterol; HDL-C, high-density lipoprotein cholesterol; TG, triglycerides; HCY, homocysteine.

**Table 5:** Multiple linear regression analysis for the relationship between CFPWV and study variables among the entire study group, excluded variables by multiple linear regression analysis in all subjects.

Characteristics	Beta in	*t*	*p* value
SBP (mmHg)	−0.473	−0.328	0.743
UA (*μ*mol/L)	0.072	1.665	0.097
TC (mmol/L)	−0.044	−1.050	0.295
HDL-C (mmol/L)	−0.060	−1.344	0.180
LDL-C (mmol/L)	−0.082	−1.950	0.052
TG (mmol/L)	0.056	1.283	0.200
HCY (*μ*mol/L)	−0.006	−0.136	0.892
Log NT-proBNP	−0.044	−0.957	0.339
Creatinine (*μ*mol/L)	0.058	1.384	0.167

CFPWV, carotid-femoral pulse wave velocity; BMI, body mass index; DBP, diastolic blood pressure; FPG, fasting plasma glucose; UAE, urine albumin (microalbumin) excretion; CI, confidence interval; SBP, systolic blood pressure; UA, uric acid; TC, cholesterol; LDL-C, low-density lipoprotein cholesterol; HDL-C, high-density lipoprotein cholesterol; TG, triglycerides; HCY, homocysteine.

## Data Availability

No additional data are available.
